# Risk of in-hospital complications after radical cystectomy for urinary bladder carcinoma: population-based follow-up study of 7608 patients

**DOI:** 10.1111/bju.12239

**Published:** 2013-07-26

**Authors:** Mieke Hemelrijck, Andreas Thorstenson, Philip Smith, Jan Adolfsson, Olof Akre

**Affiliations:** Division of Cancer Studies Cancer Epidemiology Group School of Medicine, King's College LondonLondon, UK; * Department of Molecular Medicine and Surgery, Karolinska InstituteStockholm, Sweden; † Department of Surgery Section of Urology, Capio S:t Görans HospitalStockholm, Sweden; ‡ Surgical Department, Danderyd HospitalStockholm, Sweden; § CLINTEC Department, Karolinska InstituteStockholm, Sweden; ¶ Clinical Epidemiology Unit Department of Medicine (Solna), Karolinska InstituteStockholm, Sweden

**Keywords:** bladder cancer, cystectomy, complication

## Abstract

**Objective:**

- To evaluate the risk of different in-hospital complications for patients undergoing a radical cystectomy (RC), as limited nationwide population data on short- and long-term complications after RC is available, despite it being the standard treatment for localised muscle-invasive urinary bladder cancer (UBC).

**Patients and Methods:**

- In all, 7608 persons underwent a RC after UBC diagnosis, as registered in the Swedish National Patient Register between 1964 and 2008.
- We estimated the frequency and incidences and calculated hazard ratios (HR) and 95% confidence intervals (CI) using multivariate Cox proportional hazards models.

**Results:**

- Urinary tract infection/septicaemia was the most common complication following radical cystectomy, with an incidence of 90.4 per 1,000 person years.
- There was a higher risk of urinary tract infection among patients who had a continent cutaneous reservoir (HR: 1.11 (0.94–1.30) or orthotopic neobladder 1.21 (1.05–1.39) than among those with ileal conduit.
- Similarly, continent cutaneous reservoir and orthotopic neobladder were associated with increased risks for wound and abdominal wall hernias, stones in the urinary tract, hydronephrosis and nephrostomy tube treatment, and kidney failure.
- In contrast, risk of bowel obstruction was lower among those with orthotopic neobladder than those with ileal conduit (HR: 0.64 (0.50–0.81)) and those with continent cutaneous reservoir (HR: 0.92 (0.73–1.16).

**Conclusions:**

- In-hospital complications after RC are numerous and continue to accumulate for many years after surgery, indicating the need for life-long follow-up of these patients.
- Comparison between different types of diversion should, however, be made with care because of potential confounding by indication.

## Introduction

Radical cystectomy (RC) for localised muscle-invasive urinary bladder cancer (UBC) 1 is a complex and multifaceted surgical procedure and the short- and long-term complications are well studied in single-centre series 2–12. However, limited information is available from population-wide samples providing an estimate for the general population 13.

Using a single-centre series of patients who underwent RC and urinary diversion with an orthotopic neobladder (1540 patients), Hautmann et al. 7 reported that 587 (58%) had at least one complication ≤90 days after the surgery. In another series of 358 consecutive patients who underwent RC for non-metastatic UBC, it was shown that 49% of patients had complications ≤90 days after the surgery 4. A Japanese study based on 1057 patients who underwent RC with a urinary diversion using ileum or colon showed that 61% of the patients had complications directly attributable to the urinary diversion performed, with a mean of 2.3 complications per patient 14. Another study focusing on long-term complications based on 75 patients who underwent RC and orthotopic ileal neobladder substitution showed that complications occurred in 23 patients and three died from pulmonary embolism 8.

To our knowledge there are only few nationwide reports on complications published to date. The study based on 50 625 patients from the USA Nationwide Inpatient Sample who underwent RC for UBC between 2001 and 2008 showed that the proportion of patients with in-hospital complications remained stable at 28.3% in 2001–2002 compared with 28.0% in 2007–2008. In-hospital mortality was also unchanged from 2.4% in 2001–2002 compared with 2.3% in 2007–2008 13–15. Using data from the Swedish Bladder Cancer Register (1997–2002), a follow-up questionnaire for RC was distributed to all patients treated with RC ≤3 months after diagnosis of primary UBC without distant metastasis. This study reported a reoperation rate of 24%, which was higher for patients receiving a continent cutaneous diversion 16.

We used data on all patients who underwent RC for UBC as registered in the Swedish National Patient Register from 1964 to 2008 to evaluate the countrywide burden of in-hospital complications after RC.

## Patients and Methods

To assess the occurrence of hospitalisation due to complications after RC, we selected all men and women who underwent RC due to UBC in Sweden between 1964 and 2008 (7608 patients). Information on the diagnosis of UBC was taken from the National Cancer Register 17, whereas information about the RC was taken from the National Patient Register 18. The latter register was also used to collect details on pre-specified complications. In addition, we obtained information on cause of death from the National Cause of Death Register. Information from these three registers was linked using the unique personal identity numbers.

The Swedish National Cancer Register, which is managed by the National Board of Health and Welfare, was founded in 1958 17. All incident cases of cancer in Sweden must be reported to the cancer register separately by the physician responsible and the respective pathologist/cytologist. Record linkage by means of the personal identity number ensures that each tumour is only registered once 19–20. The National Patient Register includes information about in-patient care since 1964 in different Swedish regions. The number of participating counties increased over time and the Register became nationwide in 1987. Each observation contains information on dates of admission and discharge, hospital department, codes for surgical procedures performed during the stay, and main and contributory discharge diagnoses coded according to International Classification of Diseases (ICD) 18. Since 1953, the National Board of Health and Welfare has kept a database containing cause of death data 18.

The patients who underwent RC were subdivided based on the specific urinary diversion used: ileal conduit, continent cutaneous reservoir or orthotopic neobladder. It is important to note that, especially in the beginning of the study period, ileal conduit was the most common procedure so that diversion type was often not recorded. Frequencies by diversion type are reported for the entire study period, but all analyses were based on those RCs performed as of 1990 to allow for comparisons between procedures conducted according to a similar surgical practice. As our study is based on inpatient data, we pre-defined the following complications requiring in-hospital care: bowel obstruction, UTI/septicaemia, inguinal hernia, nephrostomy tube, wound/abdominal wall hernia, stones in the urinary tract, hydronephrosis, and kidney failure (Appendix 1). All complications were defined by using standardised surgical codes and the ICD, including both primary and secondary diagnoses, using in-hospital data so that we could not use the Clavien score, which focuses on short-term complications to which our data are not sensitive. Finally, we also assessed all-cause mortality.

**Appendix 1:** Surgical and inpatient code for different complications studied

Complication	Surgical Code	Diagnosis Code (ICD9-10)
Bowel obstruction	4770; JFB 00; 4630	K562; K563; K564; K565; K566; K567; 560B; 560W; 560X; 560.10; 560.96
UTIs		N390; A410; A411; A412; A414; A415; A419; A418; N390X; N300; N309; N136; 599A; 595C; 595.00; 595.09; 590B; 590.10; 599.02; 590.00; 595X; 595A; 595W; 590A; 038X; 038E; 038B; 599.11; 599.02
Inguinal hernia	4200; JAB30; JAB10	550,99; 550X, K409
Nephrostomy tube	TKA 10; 6006; 6005	
Wound hernia	4240; JAD30; JAD10	K439; 553C; 551,21
Stones in the urinary tract	6360; JFK10; KCE02; 6008; 6269; 6202; KBJ70; 6013	592A; 592B; N201; N202; N210; N200; 594X; N218; N209
Hydronephrosis due to ureteric stricture		N133, N131; 591X; 591; N135; 593D, 593E; N130, N139; 591.99
Kidney failure		N189; 585X; N179; 586X; 729.99; 584X; N180; N199

For statistical analyses, frequency counts by diversion type were given for the entire study period, but all the following analyses were only performed for those RCs performed as of 1990. We calculated the incidence per 1000 person-years (py) for each complication after all RCs combined, as well as for the different types of urinary diversion. These incidences are thus defined by person-years for the at risk RC population. The risk of developing each of these complications was modelled using multivariate Cox-proportional hazards models in which we adjusted for age, sex, and lymph node dissection (LND). Risks were also estimated for the different types of urinary diversions used and by sex. Follow-up time was defined from the time of RC until date of complication, death, or end of study (31 December 2008), whichever came first. In summary, all frequency counts involved all adverse events for every person, whether Cox proportional hazards models were always estimated by using the first event of the studied outcome for every person. Follow-up thus varied per person depending on type of complication studied.

As the choice of type of urinary diversion is associated with UBC severity, competing risks may be involved in the analysis of complications after RC. Patients with severe disease are more likely to die early of their UBC, so that they never develop potential long-term complications. We therefore repeated the above described Cox proportional hazards models using Fine and Gray's analysis 21. This analysis takes into account that a patient is at risk of more than one mutually exclusive event, such as death from UBC, which may then prevent any other event (i.e. adverse events) from ever happening 21. Finally, the risk of developing different types of complications after RC was also shown using cumulative incidence graphs.

Statistical analyses were performed with Statistical Analysis Systems (SAS) release 9.1.3 (SAS Institute, Cary, NC). The study was approved by the Regional Ethics Committee of Stockholm (Nr 2006/1449-31/4).

## Results

In all, 7608 RCs were performed due to UBC between 1964 and 2008 in Sweden. The median (range) follow-up was 3.0 (0.0–39.7) years. About 15% of the patients also underwent a LND at the time of RC; 62% of patients had an ileal conduit, 7% had a continent cutaneous reservoir and 10% had an orthotopic neobladder. Three quarters of the RCs were performed on men and the mean age at surgery was 66 years. Baseline characteristics of the study population are shown in Table 1.

**Table 1:** Baseline characteristics of the study population by year in which the RC was performed

	1964–2008	<1980	1980–1990	1990–2000	2000–2008
N	7608	748	1724	2341	2795
N (%):					
RC:					
With ileal conduit	4727 (62.1)	519 (69.4)	1290 (74.8)	1187 (50.7)	1731 (61.9)
With continent cutaneous reservoir	546 (7.2)	0 (0.00)	6 (0.4)	283 (12.1)	257 (9.2)
With orthotopic neobladder	762 (10.0)	7 (0.9)	24 (1.4)	275 (11.8)	456 (16.3)
Undefined urinary diversion	1573 (20.7)	222 (29.7)	404 (23.4)	596 (25.5)	351 (12.6)
LND	1122 (14.7)	2 (0.3)	93 (0.5)	383 (3.6)	644 (23.0)
Mean (sd):					
Age, years	66.0 (9.1)	61.3 (9.0)	64.4 (8.0)	66.5 (9.2)	68.0 (9.2)
With ileal conduit	67.29 (8.78)	61.34 (8.52)	64.58 (7.78)	68.57 (8.46)	70.20 (8.31)
With continent cutaneous reservoir	61.77 (8.90)	57.60 (15.53)	60.80 (8.11)	62.23 (8.58)	61.09 (7.71)
With orthotopic neobladder	61.46 (8.15)	57.60 (15.53)	60.80 (8.11)	62.23 (8.58)	61.09 (7.71)
Undefined urinary diversion	65.95 (9.51)	61.33 (0.77)	64.05 (8.51)	66.63 (9.08)	69.64 (9.38)
Time between UBC diagnosis and RC, years	1.34 (2.9)	1.07 (2.1)	1.42 (2.9)	1.40 (3.0)	1.31 (3.0)
N (%):					
Sex:					
Men	5711 (75.1)	572 (76.5)	1324 (76.8)	1741 (74.4)	2074 (74.2)
Women	1897 (24.9)	176 (23.5)	400 (23.2)	600 (25.6)	721 (25.8)
Death	5471 (71.9)	704 (94.1)	1527 (88.6)	1782 (76.1)	1458 (52.2)
Median (range) follow-up, years	3.0 (0.0–39.7)	5.9 (0.0–39.7)	5.6 (0.0–29.0)	3.9 (0.0–19.0)	2.0 (0.0–9.0)
<90 days, n (%)	392 (5.2)	52 (7.0)	75 (4.4)	114 (4.9)	151 (5.4)
≥90 days, n (%)	7216 (94.9)	696 (93.0)	1649 (95.7)	2227 (95.1)	2644 (94.6)

At the beginning of the series the patients were younger with a mean age of 61.3 years before 1980, whereas the proportion of older patients increased over time resulting in a mean age of 68.0 years for the period between 2001 and 2008 (Table 1). The number of procedures also increased over time and as of 1988 LND was also performed, with ≈23% of patients undergoing LND between 2000 and 2008. Ileal conduit was the standard procedure before 1990, but the proportion of urinary diversions with orthotopic neobladder increased from 1% (before 1980) to 16% (between 2000 and 2008). The number of patients with continent cutaneous reservoir slightly decreased from 12.1% (1990–2000) to 9.2% (2000–2008) (Table 1).

Table 2 shows the frequency of in-hospital complications for those RCs performed between 1990 and 2008. UTI/septicaemia was the most common complication after RC with an incidence of 127.30 per 1000 py overall, but the highest incidence was observed for those with an orthotopic neobladder (139.62 per 1000 py). Hospitalisation for kidney failure and bowel obstruction were the second most common complications, with the highest incidence for bowel obstruction in patients with an ileal conduit (59.13 per 1000 py) and the highest incidence for kidney failure in patients with a continent cutaneous reservoir (55.92 per 1000 py) (Table 2).

**Table 2:** Frequency and incidence of complications after RC and urinary diversion, 1990–2008

	All (N = 5136)		With ileal conduit (N = 2918)		With continent cutaneous reservoir (N = 540)		With orthotopic neobladder (N = 731)		Undefined urinary diversion (N = 947)	
	N(%)	Incidence per 1000 py	N(%)	Incidence per 1000 py	N(%)	Incidence per 1000 py	N(%)	Incidence per 1000 py	N(%)	Incidence per 1000 py
Bowel obstruction:		50.78		59.13		52.51		31.96		48.76
0	4377 (85.22)		2497 (85.57)		445 (82.41)		647 (88.51)		788 (83.21)	
1	569 (11.08)		319 (10.93)		66 (12.22)		63 (8.62)		121 (12.78)	
2	115 (2.24)		54 (1.85)		22 (4.07)		13 (1.78)		26 (2.75)	
3+	75 (1.46)		48 (1.64)		7 (1.30)		8 (1.09)		12 (1.27)	
UTI/septicaemia:		127.30		130.61		131.61		139.62		107.12
0	3465 (67.46)		2040 (69.91)		338 (62.59)		440 (60.19)		647 (68.32)	
1	1043 (20.31)		565 (19.36)		114 (21.11)		167 (22.85)		197 (20.80)	
2	369 (7.18)		203 (6.96)		50 (9.26)		68 (9.30)		48 (5.07)	
3+	259 (5.04)		110 (3.77)		38 (7.04)		56 (7.66)		55 (5.81)	
Inguinal hernia:		11.15		11.25		5.80		15.46		10.75
0	4982 (97.00)		2842 (97.40)		528 (97.78)		696 (95.21)		916 (96.73)	
1	90 (1.75)		48 (1.64)		7 (1.30)		19 (2.60)		16 (1.69)	
2	42 (0.82)		15 (0.51)		5 (0.93)		11 (1.50)		11 (1.116)	
3+	22 (0.43)		13 (0.45)		0 (0.00)		5 (0.68)		4 (0.42)	
Nephrostomy tube:		17.28		17.25		16.02		20.96		15.18
0	4858 (94.59)		2772 (95.00)		511 (94.63)		675 (92.34)		900 (95.04)	
1	204 (3.97)		112 (3.84)		18 (3.33)		42 (5.75)		32 (3.38)	
2	53 (1.03)		28 (0.96)		6 (1.11)		7 (0.96)		12 (1.27)	
3+	21 (0.41)		6 (0.21)		5 (0.93)		7 (0.96)		3 (0.32)	
Hydronephrosis due to ureteric stricture:		39.89		38.74		52.17		43.49		32.05
0	4697 (91.45)		2709 (92.84)		477 (88.33)		644 (88.10)		867 (91.55)	
1	248 (4.83)		111 (3.80)		32 (5.93)		54 (7.39)		51 (5.39)	
2	99 (1.93)		51 (1.75)		16 (2.96)		16 (2.19)		16 (1.69)	
3+	92 (1.79)		47 (1.61)		15 (2.78)		17 (2.33)		13 (1.37)	
Wound hernia/hernia of the abdominal wall:		21.49		21.77		24.21		22.00		18.77
0	4859 (94.61)		2784 (95.41)		495 (91.67)		686 (93.84)		894 (94.40)	
1	163 (3.17)		77 (2.64)		28 (5.19)		25 (3.42)		33 (3.48)	
2+	114 (2.22)		57 (1.95)		17 (3.15)		20 (2.72)		20 (2.11)	
Stones in the urinary tract		28.70		19.74		52.51		37.46		27.41
0	4772 (92.91)		2781 (95.31)		457 (84.63)		663 (90.70)		871 (91.97)	
1	241 (4.69)		106 (3.63)		49 (9.07)		33 (4.51)		53 (5.60)	
2	65 (1.27)		17 (0.58)		22 (4.07)		18 (2.46)		8 (0.84)	
3+	58 (1.13)		14 (0.48)		12 (2.22)		17 (2.33)		15 (1.58)	
Kidney failure:		53.15		54.88		55.92		43.22		55.46
0	4706 (91.63)		2677 (91.74)		491 (90.93)		677 (92.61)		861 (90.92)	
1	430 (8.37)		241 (8.26)		49 (9.07)		54 (7.39)		86 (9.08)	
Death:		145.07		181.62		98.19		84.88		138.96
No	1896 (36.92)		949 (32.52)		252 (46.67)		677 (92.61)		288 (30.41)	
Yes	3240 (63.08)		1969 (67.48)		288 (53.33)		54 (7.39)		659 (69.59)	

When studying the risk of different in-hospital complications by type of diversion we found a higher risk of UTI among patients who had an orthotopic neobladder (hazard ratio [HR] 1.21, 95%CI 1.05–1.39) than in those with ileal conduit (Table 3). In contrast, these patients had a lower risk of bowel obstruction compared to those with ileal conduit (HR 0.64, 95%CI 0.50–0.81). Patients with a cutaneous reservoir were at higher risk of hydronephrosis, wound hernia, and stones than those with ileal conduit (e.g. HR wound hernia 1.44, 95%CI 1.01–2.06). Also those with an orthotopic neobladder were at increased risk of hydronephrosis than those with ileal conduits (HR 1.31, 95%CI 1.00–1.71). These patterns were also seen when stratifying by sex (Table 3). The risk of death was highest for those with ileal conduits as the HR for all other types of diversion was <1 (e.g. HR orthotopic neobladder 0.60, 95%CI 0.53–0.68). Taking into account competing risks of death using Fine and Gray analysis did not substantially alter the findings in Table 3 (Results not shown).

**Table 3:** HRs and 95% CIs for the association between different types of RC and complications and death. The analysis is restricted to procedures performed in 1990 onwards. All models are adjusted for age, gender, and LND

	HR (95% CI)		
	All (*n* = 5136)	Men (*n* = 3815)	Women (*n* = 1321)
Bowel obstruction			
With ileal conduit	1.00 (Ref)	1.00 (Ref)	1.00 (Ref)
With continent cutaneous reservoir	0.92 (0.73–1.16)	1.03 (0.77–1.37)	0.74 (0.50–1.10)
With orthotopic neobladder	0.64 (0.50–0.81)	0.67 (0.51–0.87)	0.54 (0.27–1.08)
Undefined urinary diversion	1.00 (0.83–1.20)	1.03 (0.82–1.29)	0.96 (0.70–1.32)
UTI/septicaemia			
With ileal conduit	1.00 (Ref)	1.00 (Ref)	1.00 (Ref)
With continent cutaneous reservoir	1.11 (0.94–1.30)	1.15 (0.94–1.39)	0.94 (0.71–1.26)
With orthotopic neobladder	1.21 (1.05–1.39)	1.21 (1.04–1.41)	1.54 (1.05–2.28)
Undefined urinary diversion	0.90 (0.79–1.03)	0.97 (0.83–1.13)	0.75 (0.58–0.97)
Inguinal hernia:			
With ileal conduit	1.00 (Ref)	1.00 (Ref)	1.00 (Ref)
With continent cutaneous reservoir	0.74 (0.40–1.38)	0.84 (0.45–1.56)	*
With orthotopic neobladder	1.21 (0.79–1.84)	1.29 (0.84–1.98)	*
Undefined urinary diversion	1.14 (0.75–1.74)	1.18 (0.77–1.82)	*
Nephrostomy tube:			
With ileal conduit	1.00 (Ref)	1.00 (Ref)	1.00 (Ref)
With continent cutaneous reservoir	0.89 (0.59–1.34)	0.79 (0.47–1.33)	1.00 (0.48–2.06)
With orthotopic neobladder	1.22 (0.88–1.70)	1.29 (0.91–1.82)	0.82 (0.24–2.74)
Undefined urinary diversion	0.84 (0.60–1.17)	0.93 (0.64–1.35)	0.62 (0.30–1.28)
Hydronephrosis due to ureteric stricture:			
With ileal conduit	1.00 (Ref)	1.00 (Ref)	1.00 (Ref)
With continent cutaneous reservoir	1.34 (1.00–1.80)	1.22 (0.86–1.73)	1.89 (1.06–3.36)
With orthotopic neobladder	1.31 (1.00–1.71)	1.22 (0.92–1.62)	1.63 (0.78–3.93)
Undefined urinary diversion	1.01 (0.78–1.31)	0.88 (0.64–1.20)	1.50 (0.91–2.47)
Wound hernia/hernia of the abdominal wall:			
With ileal conduit	1.00 (Ref)	1.00 (Ref)	1.00 (Ref)
With continent cutaneous reservoir	1.44 (1.01–2.06)	1.71 (1.12–2.58)	0.93 (0.47–1.81)
With orthotopic neobladder	1.05 (0.74–1.51)	1.09 (0.73–1.61)	1.26 (0.52–3.07)
Undefined urinary diversion	1.07 (0.78–1.47)	1.36 (0.94–1.95)	0.51 (0.25–1.02)
Stones in the urinary tract:			
With ileal conduit	1.00 (Ref)	1.00 (Ref)	1.00 (Ref)
With continent cutaneous reservoir	2.19 (1.64–2.91)	1.81 (1.27–2.59)	3.29 (1.92–5.66)
With orthotopic neobladder	1.32 (0.97–1.79)	1.22 (0.88–1.70)	2.02 (0.86–4.75)
Undefined urinary diversion	1.29 (0.97–1.71)	1.34 (0.89–1.72)	1.56 (0.88–2.57)
Kidney failure:			
With ileal conduit	1.00 (Ref)	1.00 (Ref)	1.00 (Ref)
With continent cutaneous reservoir	1.01 (0.74–1.40)	1.00 (0.69–1.47)	0.97 (0.52–1.79)
With orthotopic neobladder	0.81 (0.60–1.11)	0.83 (0.60–1.15)	0.87 (0.31–2.44)
Undefined urinary diversion	0.91 (0.71–1.16)	0.97 (0.73–1.29)	0.74 (0.44–1.26)
Death:			
With ileal conduit	1.00 (Ref)	1.00 (Ref)	1.00 (Ref)
With continent cutaneous reservoir	0.69 (0.61–0.79)	0.67 (0.57–0.79)	0.73 (0.58–0.91)
With orthotopic neobladder	0.60 (0.53–0.68)	0.60 (0.52–0.68)	0.63 (0.43–0.91)
Undefined urinary diversion	0.88 (0.81–0.97)	0.88 (0.79–0.98)	0.90 (0.77–1.06)

* Too few events (*n* = 7) to obtain HR estimates.

**Figure 1:**
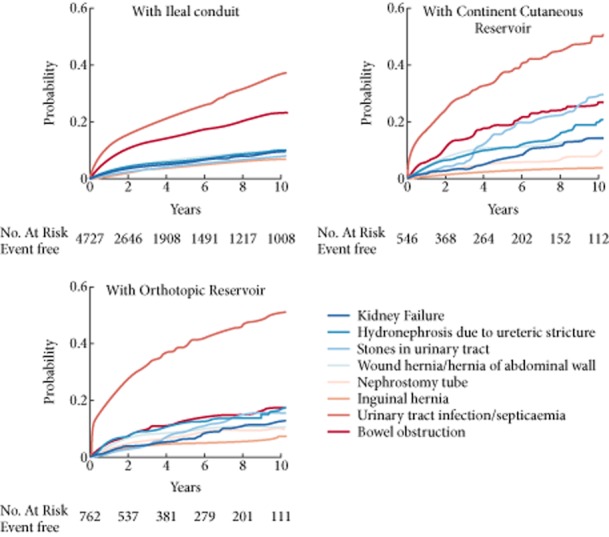
Cumulative incidence of different complications after different types of RC in Sweden, 1964–2008.

The 10-year cumulative incidence curves for complications after RC by type of urinary diversion indicate that most complications continue to accumulate during the entire period of follow-up (Fig. 1).

## Discussion

The present study is unique as it not only provides information from a population-based cohort, but also over a period of >40 years with detailed information on in-hospital complications allowing comparisons to be made between different types of diversion. It therefore enabled us to provide a general picture of complications after RC, including both low- and high-volume centres, without the risk of loss to follow-up when evaluation long-term adverse events. Our nationwide study with ≥35 years of follow-up thus showed that complications after RC are frequent and continue to accumulate many years after the surgery. About 30% of patients had UTI or septicaemia requiring hospitalisation at least once, whereas bowel obstruction occurred in almost 15% of patients. Ileal conduit was the type of urinary diversion associated with the lowest frequency of complications but also with the highest incidence of death.

Apart from the increased risk of postoperative bowel obstruction, the present data show a more preferable risk profile for ileal conduit over continent cutaneous reservoir or orthotopic neobladder. This positive risk profile is of interest given that the typical patient selection for this procedure would predict the opposite risk profile. UTI/septicaemia is a well-known complication after RC but the present data showed a lower incidence for patients with ileal conduit (130.61 per 1000 py) than for patients with an orthotopic neobladder (139.62 per 1000 py) or a continent cutaneous reservoir (131.61 per 1000 py). In contrast to those undergoing the latter two procedures, patients undergoing a RC with ileal conduit are less exposed to residual urine or repeated catheterisations as a consequence of retention of urine inside the body. Patients with an orthotopic neobladder had less bowel obstruction than patients with an ileal conduit, which may be attributed to the presence of an intestinal stoma through the abdominal wall in the latter patients. The surgically created passage of intestine through the abdominal wall is a well-known site of herniation and rotation causing bowel obstruction. Moreover, a higher rate of locally advanced disease may contribute to the higher incidence of bowel obstruction among patients who underwent a RC with ileal conduit.

A slightly increased risk of complications among patients with continent urinary diversions could be outweighed by a better health-related quality of life. In contrast to an ileal conduit, an orthotopic neobladder replicates the physiological voiding function and may seem intuitively attractive to both the surgeon and the patient. However, studies of quality-of-life outcomes have consistently failed to show any clear advantages of an orthotopic neobladder 22–25. In fact, a recent study by Anderson et al. 25 showed better patient-reported outcomes associated with ileal conduit. Nevertheless, preoperative expectations may differ and the interpretation of data is complex, while randomised studies equalising the baseline characteristics are lacking. We conclude that the present findings underscore how the intuitive attractiveness of an orthotopic neobladder could be deceiving and that it is crucial for patients to be carefully informed before the type of diversion is chosen.

The proportion of patients undergoing a LND was surprisingly low. We expected the frequency to be higher than 23% during the 2000s, but we suspect that the code for lymphadenectomy may have been underreported using hospital discharge data. As RCs historically have been decentralised to many low-volume hospitals, it is, however, expected that the number of lymphadenectomies is low compared with reports of case series from single centres of excellence.

The unique infrastructure of nationwide registers in Sweden enabled us to follow a large population-based cohort of patients for up to almost 40 years. These patients represent the entire population, thus avoiding selection bias. The validity of the Patient Register is known to be high for specific diagnoses such as septicaemia and hernia 26. However, it should be noted that because we based the present study on inpatient data, we only estimated the incidence of complications based on the records of those admitted to hospital, so that the incidence of several conditions, e.g. infections, will be underestimated as many patients are also treated as outpatients. Another limitation is the lack of detailed information on clinical characteristics, e.g. cancer stage and grade, the extent of LND, and specific operative techniques as well as the surgical skills (i.e. learning curve) and hospital volume. Therefore, we cannot evaluate the use of specific devices, e.g. anti-reflux nipples, or the choice of colon or ileum for the diversion. Comparing different urinary diversions in terms of in-hospital complications is complex because the choice of diversion may be influenced by the perceived baseline risk of such outcomes, a phenomenon known as confounding by indication. Ileal conduit, for instance, is preferred among the elderly, those with more advanced disease stage and more comorbidity. Despite the likely higher baseline risk of complications among patients with ileal conduit, we found that ileal conduits were negatively associated with several complications. Moreover, Fine and Gray analyses indicated that competing risks did not play a major role in these analyses.

Whereas oncological surveillance can be terminated 5 years after RC 27, these data thus underscore the need for a continuing follow-up of functional outcomes and complications. At present, the European Association of Urology guidelines recommend that such surveillance may be stopped after 15 years, but the present data as well as others 28 do not indicate any time point beyond which complications do not continue to accumulate. We therefore suggest that functional follow-up of these patients be lifelong 27. In particular, surveillance of renal function seems warranted, and a liberal use of imaging techniques on demand to perhaps prevent the development of kidney failure in a proportion of the patients.

In conclusion, overall the present data show that complications after RC and urinary diversion are numerous and continue to accumulate for many years after surgery, suggesting that follow-up of these patients should be lifelong. When comparing the frequency of complications between different types of urinary diversion, ileal conduit was associated with more events of bowel obstruction, but fewer instances of other complications, e.g. infections and hydronephrosis.

## Abbreviations

HR: hazard ratio
LND: lymph node dissection
py: person-years
RC: radical cystectomy
UBC: urinary bladder cancer

## References

[b1] Stenzl A, Cowan NC, De Santis M (2012). [Treatment of muscle-invasive and metastatic bladder cancer: update of the EAU Guidelines]. Actas Urol Esp.

[b2] Maffezzini M, Campodonico F, Canepa G, Gerbi G, Parodi D (2008). Current perioperative management of radical cystectomy with intestinal urinary reconstruction for muscle-invasive bladder cancer and reduction of the incidence of postoperative ileus. Surg Oncol.

[b3] Hautmann RE, de Petriconi RC, Volkmer BG (2010). Lessons learned from 1,000 neobladders: the 90-day complication rate. J Urol.

[b4] Novara G, De Marco V, Aragona M (2009). Complications and mortality after radical cystectomy for bladder transitional cell cancer. J Urol.

[b5] Chang SS, Cookson MS, Baumgartner RG, Wells N, Smith JA (2002). Analysis of early complications after radical cystectomy: results of a collaborative care pathway. J Urol.

[b6] Kulkarni JN (2011). Perioperative morbidity of radical cystectomy: a review. Indian J Urol.

[b7] Hautmann RE, de Petriconi RC, Volkmer BG (2011). 25 years of experience with 1,000 neobladders: long-term complications. J Urol.

[b8] Hadzi-Djokic J, Pejcic T, Vuksanovic A, Acimovic M, Dzamic Z (2007). Orthotopic neobladder: a 22-year experience. Acta Chir Iugosl.

[b9] Eswara JR, Efstathiou JA, Heney NM (2012). Complications and long-term results of salvage cystectomy after failed bladder sparing therapy for muscle invasive bladder cancer. J Urol.

[b10] Smith AB, Raynor MC, Pruthi RS (2011). Peri- and postoperative outcomes of robot-assisted radical cystectomy (RARC). BJU Int.

[b11] Ramani VA, Bromage SJ, Clarke NW (2009). A contemporary standard for morbidity and outcome after radical cystectomy. BJU Int.

[b12] Clark PE, Stein JP, Groshen SG (2005). Radical cystectomy in the elderly: comparison of clincal outcomes between younger and older patients. Cancer.

[b13] Kim SP, Boorjian SA, Shah ND (2012). Contemporary trends of in-hospital complications and mortality for radical cystectomy. BJU Int.

[b14] Shimko MS, Tollefson MK, Umbreit EC, Farmer SA, Blute ML, Frank I (2011). Long-term complications of conduit urinary diversion. J Urol.

[b15] Konety BR, Allareddy V, Herr H (2006). Complications after radical cystectomy: analysis of population-based data. Urology.

[b16] Liedberg F, Holmberg E, Holmang S (2012). Long-term follow-up after radical cystectomy with emphasis on complications and reoperations: a Swedish population-based survey. Scand J Urol Nephrol.

[b17] The National Board of Health and Welfare (2011). http://www.socialstyrelsen.se/register/halsodataregister/cancerregistret.

[b18] http://www.socialstyrelsen.se/en/Statistics/Statistical_databases.htm.

[b19] Mattsson B, Wallgren A (1984). Completeness of the Swedish Cancer Register. Non-notified cancer cases recorded on death certificates in 1978. Acta Radiol Oncol.

[b20] http://www.socialstyrelsen.se/en/Statistics/statsbysubject/Cancer+Registry.htm.

[b21] Bakoyannis G, Touloumi G (2012). Practical methods for competing risks data: a review. Stat Methods Med Res.

[b22] Porter MP, Penson DF (2005). Health related quality of life after radical cystectomy and urinary diversion for bladder cancer: a systematic review and critical analysis of the literature. J Urol.

[b23] Lee CT (2009). Quality of life following incontinent cutaneous and orthotopic urinary diversions. Curr Treat Options Oncol.

[b24] Gerharz EW, Mansson A, Hunt S, Skinner EC, Mansson W (2005). Quality of life after cystectomy and urinary diversion: an evidence based analysis. J Urol.

[b25] Anderson CB, Feurer ID, Large MC (2012). Psychometric characteristics of a condition-specific, health-related quality-of-life survey: the FACT-Vanderbilt Cystectomy Index. Urology.

[b26] Ludvigsson JF, Andersson E, Ekbom A (2011). External review and validation of the Swedish national inpatient register. BMC Public Health.

[b27] Gakis G, Efstathiou J, Lerner SP (2013). ICUD-EAU International Consultation on Bladder Cancer 2012: radical cystectomy and bladder preservation for muscle-invasive urothelial carcinoma of the bladder. Eur Urol.

[b28] Madersbacher S, Schmidt J, Eberle JM (2003). Long-term outcome of ileal conduit diversion. J Urol.

